# Medical evaluation of first presentation of psychotic symptoms in children and adolescents

**DOI:** 10.3389/fpsyt.2026.1678974

**Published:** 2026-04-14

**Authors:** Orly Lavan, Esther Ganelin-Cohen, Amit Goldstein, Tomer Mevorach, Shani Frank, Alan Apter, Silvana Fennig, Noa Benaroya-Milshtein, Amir Krivoy

**Affiliations:** 1 Gray School of Medicine, Faculty of Medical and Health Sciences, Tel Aviv University, Ramat-Aviv, Israel; 2 Department of Psychiatry, Schneider Children’s Medical Center of Israel, Petah Tikva, Israel; 3 Department of Neurology, Schneider Children’s Medical Center of Israel, Petah Tikva, Israel; 4 Children and Adolescent Psychiatric Clinic, Tel Aviv Sourasky Medical Center – Ichilov, Tel Aviv, Israel; 5 Geha Mental Health Center, Petah Tikva, Israel

**Keywords:** First-episode psychosis, medical evaluation, medical workup, pediatric psychiatry, pediatric psychosis

## Abstract

**Introduction:**

Psychotic symptoms in children and adolescents may represent either normative developmental phenomena or severe psychiatric and medical conditions, requiring careful differential diagnosis.

**Methods:**

This retrospective study aimed to evaluate the medical workup of children and adolescents admitted for a first presentation of psychotic symptoms at a tertiary pediatric center over a 10-year period. The sample included 68 patients (mean age 13.7 ± 3.7 years) who underwent clinician-directed evaluations including physical exams, laboratory tests, toxicology screens, neuroimaging, and lumbar puncture when indicated.

**Results:**

Sixteen patients (23.5%) were diagnosed with substance-/medication-induced or medically-associated psychosis. In this cohort, younger age, very early onset psychosis (<13 years), and catatonia at first presentation were more frequently observed among patients with secondary etiologies, whereas documented prior subthreshold symptoms were more frequently documented among those diagnosed with primary psychiatric disorders. Most investigations did not identify a secondary cause, reflecting clinician-directed evaluation in routine practice; however, selected cases (e.g., autoimmune encephalitis, multiple sclerosis) illustrate the clinical importance of careful assessment when specific suspicion is present.

**Conclusion:**

These findings suggest that targeted medical evaluation may be useful in pediatric psychosis, particularly when clinical features raise suspicion for secondary etiologies, and may help inform clinical decision-making in tertiary pediatric settings.

## 1 Introduction

Psychotic symptoms include delusions, hallucinations, and disorganized speech and behavior. Their prevalence is 17% amongst children and adolescents 9–12 years old (y/o) and 7.5% among those 13–18 y/o (1). In youth, these symptoms lie in a continuum from developmentally appropriate to severe psychiatric illness and may also be related to an underlying medical condition (2–5). Psychotic disorders are categorized into primary and secondary disorders (6). Although most patients with psychosis are diagnosed with a primary psychiatric disorder, mainly schizophrenia or bipolar disorder (7), elimination of non-psychiatric disorders is important and even a requirement of the Diagnostic and Statistical Manual of Mental Disorders (DSM), 5^th^ edition (8).

Evaluation of patients who present with psychotic symptoms requires a comprehensive assessment including a thorough history, physical examination, and decisions to perform laboratory tests for infectious, neurologic, autoimmune, or metabolic disease, as well as an electroencephalogram (EEG) and neuroimaging tests (9). Secondary psychotic disorders include substance-induced psychosis or psychosis due to underlying medical conditions, mainly neurologic, autoimmune, infectious, rheumatologic, endocrine, metabolic, and chromosomal disorders (4). Approximately 10% of all cases of mental health presentations in adults are attributable to these non-psychiatric disorders (10). In children and adolescents, a tertiary-care cohort study reported medical disorders likely contributing to psychosis in 12.5% of cases (11).

Among published national guidelines for the treatment of first-episode psychosis, the extent of recommended medical workup varies (12). A comparator review of the published guidelines revealed disagreements regarding the different aspects of the medical investigation recommended, highlighting the absence of information about the clinical reasoning for these investigations (13). The National Collaborating Centre for Mental Health (NICE) guidelines (14) and Canadian Psychiatric Association Guidelines (15) do not make specific recommendations for the medical workup. Instead, they suggest a baseline evaluation prior to treatment with antipsychotic medications.

According to the Practice Parameter for Assessment and Treatment of Children and Adolescents with Schizophrenia (16), laboratory testing is recommended at the time of first diagnosis, including CBC, liver and renal function, metabolic parameters, and thyroid function. More extensive evaluation is indicated only in atypical presentations, such as a gross deterioration in cognitive and motor abilities, focal neurologic symptoms, or delirium. It is important to note that unless properly investigated, these non-psychiatric disorders can present as psychosis alone, thus clinicians are unable to differentiate between these two conditions. This classification is important as secondary conditions must be managed and treated differently to be resolved.

Although clinical guidelines outline general recommendations for the evaluation of first-episode psychosis, real-world diagnostic practices and their clinical correlates in pediatric populations remain insufficiently characterized. In particular, limited data are available regarding the clinical features that differentiate primary psychiatric disorders from secondary (organic or substance-induced) causes in hospitalized children and adolescents.

The aim of the present retrospective study was therefore to characterize the real-world clinical presentation and evaluation patterns of children and adolescents admitted with psychotic symptoms to a large tertiary referral center, and to compare clinical features and workup patterns between primary psychiatric disorders and substance-/medication-induced or medically-associated psychosis.

## 2 Materials and methods

### 2.1 Study design

This retrospective study reviewed electronic records of all pediatric admissions with suspected psychosis at a tertiary center from February 2011 to May 2021. Included were 68 children and adolescents (ages 3.1–17.9) hospitalized with a first presentation of psychotic symptoms, confirmed by a board-certified child and adolescent psychiatrist. Exclusions included prior psychosis, known malignancy, or lack of psychiatrist-confirmed symptoms. All participants underwent medical investigations as clinically indicated.

### 2.2 Ethics approval

This retrospective chart-review study was approved by the Institutional Review Board of Schneider Children’s Medical Center of Israel (Approval No. 0626-20-RMC). Due to the retrospective design and the use of anonymized clinical data, the requirement for informed consent was waived by the IRB. All procedures were conducted in accordance with the principles of the Declaration of Helsinki. Access to identifiable patient information was restricted to authorized study personnel.

#### 2.1.1 Classification of psychosis

Records were reviewed for up to 12 months following the index presentation to assess diagnostic stability. The classification process followed a structured, consensus-based approach at our tertiary medical center. Each case was reviewed in a multidisciplinary case conference involving a board-certified child and adolescent psychiatrist, pediatrician, and pediatric neurologist. The team reviewed all available data, including psychiatric assessments, medical investigations performed as clinically indicated, treatment response, and follow-up documentation.

Classification of substance-/medication-induced or medically-associated psychosis required a clear temporal association between the medical condition, medication exposure, or substance use and the onset of psychotic symptoms, as well as documented improvement or resolution of symptoms following treatment of the underlying condition or discontinuation of the suspected agent. The absence of a more plausible alternative explanation was also considered essential.

Substance-/medication-induced or medically-associated psychosis was defined as psychosis attributable to an identifiable non-psychiatric cause, falling into one of the following categories:

1. general medical condition (e.g., autoimmune encephalitis, thyroid dysfunction, neurologic or infectious disease) with clinical and temporal association to the psychotic episode.
2. Psychosis induced by prescribed medications with known psychiatric side effects.
3. Psychosis triggered by substance use, as indicated by toxicology screens and clinical history.

Psychosis due to a primary psychiatric disorder included patients who, after clinically indicated medical evaluation and clinical follow-up, were diagnosed with a primary psychiatric disorder (e.g., schizophrenia, bipolar disorder, or psychotic depression) without evidence of a contributing medical or substance-related etiology.

In cases where diagnostic ambiguity existed (e.g., mild laboratory abnormalities without clear clinical significance), classification was made based on expert consensus, prioritizing the overall clinical picture and evolution over time.

### 2.2 Measures

Demographic and clinical data collected included age at hospitalization, sex, medical history, family psychiatric history, drug use, medications at admission, psychiatric history, and season at admission. Additional data included length of hospitalization (LoH) and recommended medications. Data regarding the medical workup and its results were recoded for each patient.

#### 2.2.1 Psychosis clinical characteristics

The clinical characteristics of psychosis were listed according to the psychiatric evaluation and included psychosis onset, course, and symptoms: delusions, hallucinations, disorganized speech and behavior, somatic complaints, suicidality, and aggression. The retrospective evaluation of prodromal features included positive (hallucinations and delusions) or negative (apathy, lack of drive, poverty of speech, social withdrawal, and self-neglect) attenuated symptoms and functional decline, based on the information retrieved from their medical records. Neurological and physical signs were also listed.

#### 2.2.2 Medical evaluation

The medical evaluation included blood tests (CBC, neutrophil to lymphocyte ratio (NLR), and hemoglobin level); chemistry analysis for glucose levels, kidney and liver function, C-reactive protein (CRP), creatinine phosphokinase (CPK), erythrocyte sedimentation rate (ESR), ammonia, and thyroid-stimulating hormone (TSH). Additional laboratory tests were for autoimmune antibodies (anti-streptolysin O, thyroid antibodies, celiac antibodies, autoimmune encephalitis, and serology) and metabolic tests (porphyria, organic acids, ceruloplasmin, copper, and infections). Additional clinical data collected included urine toxicology screen for drugs, electroencephalogram (EEG), fundus examination, brain CT or MRI imaging tests, and lumbar puncture.

Advanced investigations (EEG, neuroimaging, lumbar puncture) were ordered at clinician discretion in routine care and were not performed universally.

Medical history and pre-admission medications were abstracted from routine clinical records and are summarized descriptively, consistent with the granularity of documentation. Pre-admission exposures are stated separately from in-hospital treatments to avoid ambiguity; relevant summaries appear in **Table 1**.

**Table 1:** Demographic and clinical characteristics of study population (n=68).

Variable		Total (n=68)	Psychosis due to a primary psychiatric disorder (n (%)=52)	Substance-/medication-induced or medically-associated psychosis (n (%)=16)	P	OR	CI
Age (mean ± SD), yrs		13.7 ± 3.7	14.3 ± 3.1	11.5 ± 4.8	**0.04**		
Sex	Male	32 (46%)	25 (48%)	7 (43%)	0.76	1.19	0.38-3.67
	Female	36 (53%)	27 (52%)	9 (56%)			
Previous Medical History (non-psychiatric)	No history	49 (72%)	40 (77%)	9 (56%)	0.18	Ref	
	*Neurologic disease	8 (12%)	6 (12%)	2 (13%)	0.66	1.48	0.25-8.57
	Non-neurological	11 (16%)	6 (12%)	5 (31%)	0.06	3.7	0.92-14.86
Medications at admission	Any drug	27 (40%)	16 (31%)	11 (69%)	**0.01**	4.95	1.47-16.60
	Antiepileptics	4 (6%)	2 (4%)	2 (13%)	0.22	3.57	0.46-27.67
	Psychotropics	15 (22%)	12 (23%)	3 (19%)	0.87	1.11	0.02-0.83
	**Other	10 (15%)	3 (6%)	7 (44%)	**0.001**	12.7	2.75-58
Illicit drugs (cannabis)		2 (3%)	1 (2%)	1 (6%)	0.39	3.4	0.2-57.6
Family psychiatric history		21 (31%)	17 (33%)	4 (25%)	0.56	0.68	0.19-2.44
Season	Winter	39 (57%)	27 (52%)	12 (75%)	0.11	1.12	0.1- 1.26
	Summer	29 (42%)	25 (48%)	4 (25%)			
Previous Psychiatric history	ADHD	13 (19%)	11 (46%)	2 (12.5%)	0.44	0.53	0.10-2.70
	Anxiety Disorder	5 (7%)	3 (6%)	2 (13%)	0.83	0.88	0.27-2.8
	Learning Disorders	4)6%)	3 (6%)	1 (6%)			
	ASD	2 (3%)	1 (2%)	1 (6%)			
	Intellectual Disability	2 (3%)	1 (2%)	1 (6%)			
	Personality Disorder	2 (3%)	2 (4%)	0			
	Total	27 (40%)	21 (40%)	6 (38%)			

*Neurologic disease: CP, epilepsy, migraine; non-neurologic disease: celiac, hearing impairment, nephrotic syndrome, PCO.

**Other: antibiotics, hypnotics, medications for high blood pressure, psychostimulants.

Bold values indicate statistically significant findings (p < 0.05).

### 2.3 Statistical analysis

Analyses were conducted using the Statistical Package for the Social Sciences, Version 20 (IBM; SPSS, Chicago, IL). Descriptive data are presented as mean ± standard deviation or rates (%). We compared two groups of patients: those with psychosis due to a primary psychiatric disorder and those with substance-/medication-induced or medically-associated psychosis. Given the relatively small sample size of the substance-/medication-induced or medically-associated psychosis group, continuous variables were compared between groups using the Mann–Whitney U test. Chi-square test or Fisher’s exact test were used as necessary to compare categorical parameters. Multivariate analysis was performed using binary logistic regression analyses, with substance-/medication-induced or medically-associated psychosis as a dependent variable controlling for covariates. A p value < 0.05 was considered to indicate statistical significance.

## 3 Results

The initial sample consisted of 122 children and adolescents admitted due to first presentation of psychotic symptoms at the hospital’s emergency department or pediatric ward. Of 54 patients excluded from the study, 33 had an initial provisional diagnosis of psychosis made by the pediatric team at admission, which was later revised following comprehensive psychiatric assessment. In these cases, the presenting symptoms, typically acute behavioral changes such as agitation, withdrawal, or disorganized behavior, were ultimately attributed to non-psychotic psychiatric conditions, including anxiety disorders, adjustment disorders, depressive episodes, or other non-psychotic presentations. These patients were therefore excluded, as they did not meet criteria for a psychotic disorder upon specialist evaluation. Seven had a known malignancy that was already diagnosed as related to their psychotic symptoms in the context of secondary delirium states, six medical records were duplicates, and three patients were transferred to another hospital with no medical workup performed. Four patients were not diagnosed at first psychotic episode, and no psychiatric evaluation was performed for one due to family refusal.

The final study group comprised 68 patients (32 boys, 36 girls) with a mean age 13.7± 3.7 (range 3.1 – 17.9) years. Their LoH was 7.3 ± 5.8 days. After a full medical workup, 16 (23.5%) patients were diagnosed with substance-/medication-induced or medically-associated psychosis and 52 (76%) with psychosis due to a primary psychiatric disorder (**Table 2**). Demographic and clinical characteristics of the study sample are presented in **Table 1**. Patients were categorized as having either psychosis due to a primary psychiatric disorder or substance-/medication-induced or medically-associated psychosis following a multidisciplinary discussion among the pediatrician, neurologist, and psychiatrist, who collectively reviewed each patient’s clinical evaluation and results of the medical workup. Variables were compared between the two groups. The mean age of the group with substance-/medication-induced or medically-associated psychosis was 11.5 ± 4.8 years, significantly lower as compared to the mean age of 14.3 ± 3.1 y/o in the psychosis due to a primary psychiatric disorder group (p=.040). Of 27 (40%) patients with a history of psychiatric disorder, the most prevalent diagnosis was ADHD (19%), followed by anxiety (7%) and learning disorders (6%). Most (72%) patients had no history of a medical illness, while 27(40%) reported taking medications. The group that reported taking no medications included more patients from the psychosis due to a primary psychiatric disorder group as compared to the substance-/medication-induced or medically-associated psychosis group, 69% and 31%, respectively (p=0.009).

**Table 2:** Diagnoses of the study sample at discharge.

Diagnostic category	Subcategory diagnosis		N (%)
Substance-/medication-induced or medically-associated psychosis	General medical condition	Autoimmune encephalitis	3 (4%)
		Multiple Sclerosis	1 (1%)
		Hashimoto thyroiditis	2 (3%)
		Epilepsy	1 (1%)
		*Other	3 (4%)
	Medication-induced Psychosis		5 (7%)
	Substance-induced Psychosis		1 (1%)
Psychosis due to a primary psychiatric disorder	Psychiatric diagnosis	Schizoaffective	3 (4%)
		Schizophrenia	10(15%)
		Affective disorders	2 (3%)
		†Other	8 (12%)
		‡Psychosis NOS	29(43%)
Total			68 (100%)

NOS, Not otherwise specified.

*Other (medical) – Obstructive Hydrocephalus, Autoimmune Hepatitis, Childhood Acute Neuropsychiatric Symptoms (CANS).

†Other (psychiatric) – anxiety disorders, conduct disorders, eating disorders, neurodevelopmental disorders.

‡Psychosis NOS – unspecified psychotic disorder.

During hospitalization, most patients (87%) received psychiatric medications, predominantly antipsychotics (76%).

### 3.1 Clinical characteristics of psychosis

**Figure 1:**
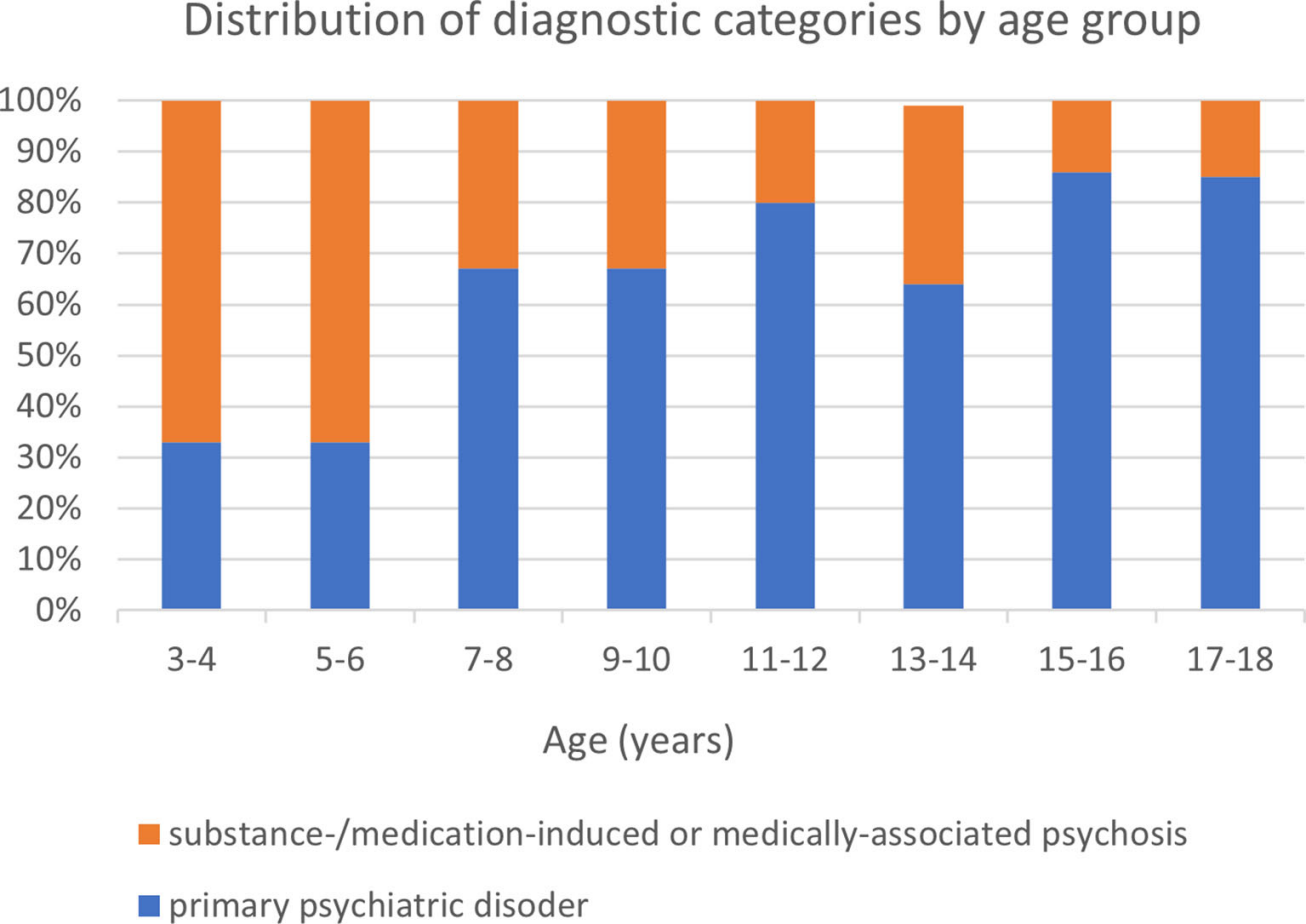
Distribution of diagnostic categories by age group. The figure illustrates the distribution of psychosis due to a primary psychiatric disorder versus substance-/medication-induced or medically associated psychosis among children and adolescents presenting with first psychotic symptoms, shown by age group.

The clinical presentation of all patients according to the evaluation of a psychiatrist is presented in **Table 3**. While 69% of patients presented with acute onset of symptoms, there were no significant differences between the groups. Early onset psychosis (<13 y/o) was identified in 20 (29%) patients with a statistically significant difference between the two groups (p<0.05). **Figure 1** shows the ratio of psychosis cases, classified by age. In a univariate analysis performed, catatonia was associated with greater risk of substance-/medication-induced or medically-associated psychosis (p=0.04, OR=5.44, CI 1.07–27.63). There were no differences between the groups regarding somatic complaints, aggression, and suicidality. Prior positive and/or negative subthreshold symptoms were documented by 71% of the psychosis due to a primary psychiatric disorder group, as compared to 44% of the substance-/medication-induced or medically-associated psychosis group (p<0.02), and were associated with a lower risk of being diagnosed with substance-/medication-induced or medically-associated psychosis (OR = 0.23, CI 0.19-13.98). However, given the retrospective design and lack of standardized assessment, this finding should be interpreted cautiously.

**Table 3:** Clinical characteristics of psychosis of the study population on admission.

Clinical characteristic		Total (n=68)	psychosis due to a primary psychiatric disorder (n=52)	substance-/medication-induced or medically-associated psychosis (n=16)	p	OR	CI
Psychosis Course	Acute	47 (69%)	34 (65%)	13 (81%)	0.23	0.43	0.11-1.73
	Chronic	21 (31%)	18 (35%)	3 (19%)			
Early Onset	≤13Y	20 (29%)	11 (21%)	9 (56%)	**0.01**	4.79	1.45-15.76
	≥13Y	48 (71%)	41 (78%)	7 (44%)			
Catatonia		7 (10%)	3 (6%)	4 (25%)	**0.04**	5.44	1.07-27.63
Hallucinations	Total	40 (59%)	32 (62%)	8 (50%)	0.41	0.62	0.2- 1.93
	Visual	21 (31%)	13 (25%)	8 (50%)	0.07		
	Auditory	24 (57%)	21 (40%)	3 (19%)	0.14		
	Tactile	2 (3%)	0	2 (13%)	0.05		
	Behavior	4 (6%)	4 (8%)	0	0.5		
Delusions		50 (73%)	37 (71%)	13 (81%)	0.42	1.75	0.43-7.0
Abnormal thought process		51 (75%)	40 (77%)	11 (69%)	0.51	0.66	0.19-2.27
prior documented positive and/or negative subthreshold symptoms	Positive	28 (41%)	23 (44%)	5 (31%)	**0.02**	0.23	0.19-13.98
	Negative	12 (18%)	11 (21%)	1 (6%)			
	Dysfunction	29 (29%)	19 (37%)	1 (6%)			
	None	24 (35%)	15 (29%)	9 (56%)			
Neurologic/physical signs	Seizures	5 (7%)	4 (8%)	1 (6%)	0.84	0.80	0.08-7.71
	Respiratory	7 (10%)	5 (10%)	2 (13%)	0.74	1.34	0.23-7.68
	Fever	12 (18%)	9 (17%)	3 (19%)	0.89	1.10	0.26-4.68
	None	50 (74%)	40 (77%)	10 (63%)	0.25	2	0.60-6.64

Bold values indicate statistically significant findings (p < 0.05).

### 3.2 Medical evaluation

Findings of the medical evaluation are summarized in **Table 4**. Physical and neurological signs included rigidity, positive Babinski reflex, and tremor. C-reactive protein (CRP) was tested in 98% of patient, with abnormal results in only 4 cases (6%). Ammonia levels were assessed in 42 patients (62%), all within normal range. Mean TSH level was 2.11 ± 1.37, with no significant group differences. ASLO antibodies were tested in 15 patients (22%), and abnormal results were found in 3—all of whom were ultimately diagnosed with psychosis due to a primary psychiatric disorder.

**Table 4A:** Medical investigation. Abnormal findings and diagnostic investigations.

Investigation	Psychosis due to a primary psychiatric disorder (n=52) Abnormal n (%)	Substance-/medication-induced or medically-associated psychosis (n=16) Abnormal n (%)	P-value
Laboratory Tests			
Complete Blood count (CBC)			
WBC (N tested=68)	2/52 (3.8%)	1/16 (6.3%)	0.56
Chemistry			
Glucose (N tested=67)	0/51 (0.0%)	0/16 (0.0%)	1
Liver enzymes (N tested=66)	6/50 (12%)	3/16 (18.8%)	0.67
Renal function (N tested=67)	1/51 (2%)	0/16 (0.0%)	1
CRP (N tested=67)	3/51 (5.9%)	1/16 (6.2%)	1
CPK (N tested=24)	4/19 (21.1%)	1/5 (20%)	1
ESR (N tested=19)	0/14 (0.0%)	1/5 (20%)	0.26
Ammonia (N tested=42)	0/33 (0.0%)	0/9 (0.0%)	1
TSH (N tested=56)	5/44 (11.4%)	1/12 (8.3%)	1
Autoimmune Antibodies			
ASLO (N tested=15)	3/12 (25.0%)	0/3 (0.0%)	1
Serology (N tested=45)	5/36 (13.9%)	3/9 (33.3%)	0.32
Thyroid Ab (N tested=26)	1/19 (5.3%)	2/7 (28.6%)	0.17
Celiac (N tested=5)	0/3 (0.0%)	1/2 (50.0%)	0.4
Autoimmune Encephalitis (N tested=16)	0/9 (0.0%)	0/7 (0.0%)	1
Metabolic tests			
Porphyria (N tested=21)	0/17 (0.0%)	0/4 (0.0%)	1
Organic Acids (N tested=14)	0/11 (0.0%)	0/3 (0.0%)	1
Ceruloplasmin (N tested=41)	6/34 (17.6%)	0/7 (0.0%)	0.56
Copper (N tested=24)	3/22 (13.6%)	0/2 (0.0%)	1
Infections (N tested=28)	3/23 (13.0%)	0/5 (0.0%)	1
Additional Tests			
Urine Toxicology (N tested=25)	0/19 (0.0%)	1/6 (16.7%)	0.24
Eye Examination (N tested=55)	0/42 (0.0%)	1/13 (7.7%)	0.23
CT (N tested=11)	2/6 (33.3%)	0/5 (0.0%)	0.45
MRI (N tested=45)	10/34 (29.4%)	7/11 (63.6%)	0.07
EEG (N tested=42)	3/30 (10.0%)	1/12 (8.3%)	1.00
LP (N tested =22)	3/14 (21.4%)	5/8 (62.5%)	0.08

**Table 4B:** Quantifiable laboratory values.

Laboratory value	Psychosis due to a primary psychiatric disorder Mean ± SD	Substance-/medication-induced or medically-associated psychosis Mean ± SD	Total Mean ± SD	MW p-value
WBC	8.30 ± 3.02 (n=51)	7.83 ± 2.38 (n=16)	8.19 ± 2.87 (n=67)	0.276
Hemoglobin	13.76 ± 1.29 (n=51)	13.16 ± 1.23 (n=16)	13.61 ± 1.29 (n=67)	0.101
NLR	2.85 ± 2.71 (n=51)	2.59 ± 3.13 (n=16)	2.79 ± 2.79 (n=67)	0.106
CPK	487.44 ± 971.42 (n=16)	127.50 ± 69.62 (n=6)	389.27 ± 837.92 (n=22)	0.802
Ammonia	46.97 ± 19.06 (n=33)	52.33 ± 18.42 (n=9)	48.04 ± 18.85 (n=42)	0.459
TSH	2.11 ± 1.37 (n=46)	2.55 ± 2.39 (n=11)	2.202 ± 1.60 (n=57)	0.903
Glucose	97.33 ± 21.99 (n=51)	93.27 ± 17.19 (n=15)	96.41 ± 20.94 (n=66)	0.582
Ceruloplasmin	19.38 ± 5.61 (n=26)	22.60 ± 8.65 (n=5)	19.90 ± 6.14 (n=31)	0.129
Copper	103.18 ± 22.46 (n=17)	124 ± 1.41 (n=2)	105.37 ± 22.17 (n=19)	0.187

*N tested* denotes the number of patients who underwent each investigation. Abnormal/positive findings are reported as *n/N tested (%)*. Continuous laboratory values are presented as mean ± SD with test-specific *n* and compared between groups using the Mann–Whitney U test. Categorical comparisons were performed using Fisher’s exact test (or Chi-square where appropriate). Sample sizes vary by test because investigations were ordered as part of routine clinical care and were not performed uniformly in all patients.CBC, complete blood count; WBC, white blood cell count; NLR, neutrophil-to-lymphocyte ratio; CRP, C-reactive protein; CPK, creatine phosphokinase; ESR, erythrocyte sedimentation rate; TSH, thyroid-stimulating hormone; ASLO, antistreptolysin O; CT, computed tomography; MRI, magnetic resonance imaging; EEG, electroencephalography; LP, lumbar puncture; MW, Mann–Whitney U test.

MRI was performed in 45 patients (66%), with abnormal findings observed in 17 cases (38%). These included non-specific white matter lesions, pineal cysts, and mild ventricular enlargement. Among these, only 3 findings were deemed clinically relevant and contributed to the diagnosis of medically associated psychosis. Relevant abnormalities included demyelinating lesions and ventricular enlargement, which supported diagnoses such as multiple sclerosis and autoimmune encephalitis. The interpretation of MRI results was performed as part of a multidisciplinary clinical evaluation. Incidental or non-specific findings were not considered sufficient for diagnostic classification.

Lumbar puncture was performed in 22 patients (32%), with abnormal results observed in 7 cases (32%). Clinically relevant findings included the presence of intrathecal antibodies against neuronal surface antigens, such as NMDA receptor antibodies, and elevated protein levels. These findings led to diagnoses including autoimmune encephalitis and multiple sclerosis. Based on the clinical presentation and additional supporting investigations, 4 of the 7 cases were classified as substance-/medication-induced or medically associated psychosis. The remaining 3 abnormal results were considered incidental or of uncertain clinical significance and did not contribute to diagnostic classification. Among the patients classified as having medically associated psychosis, several cases demonstrated clear diagnostic findings. These included one case of NMDA receptor encephalitis confirmed by positive CSF antibodies, one case of multiple sclerosis with demyelinating lesions on MRI and positive oligoclonal bands in CSF, and one case of obstructive hydrocephalus diagnosed based on ventricular dilation in neuroimaging. These findings were considered directly relevant to the patients’ psychotic symptoms and led to a reclassification as medically-associated psychosis.

## 4 Discussion

In this retrospective real-world study of children and adolescents hospitalized with psychotic symptoms in a tertiary medical center, we characterized the clinical presentation, evaluation patterns, and final etiologies observed in routine practice. The medical workup was clinician-directed and not protocol-driven, reflecting routine decision-making in a tertiary inpatient setting.

In this cohort, nearly one quarter of patients were ultimately diagnosed with a secondary etiology. Younger age and catatonia were more frequently observed among patients with secondary causes, whereas prior documented subthreshold symptoms were more common among those diagnosed with primary psychiatric disorders. These findings should be interpreted within the context of a non-standardized evaluation approach and are intended to describe patterns observed in this setting rather than to define the diagnostic yield of specific investigations.

In our cohort, 23.5% of children and adolescents hospitalized with first presentation of psychotic symptoms were classified diagnosed with substance-/medication-induced or medically-associated psychosis. Among these cases, approximately two-thirds were attributed to an underlying medical condition, nearly one-third with medication-induced psychosis, and 6% with substance-induced psychosis. Etlouba et al. reported an underlying medical condition in more than half of adult patients at first presentation of psychotic symptoms in an ER setting (17). Gianitelli et al. reported a lower rate of 12.5% of children with schizophrenia diagnosis who had organic factors explaining their diagnosis (11).Their lower rate as compared to our results is probably related to the different characteristics of their study population which only included patients with schizophrenia diagnosis.

Patients diagnosed with substance-/medication-induced or medically-associated psychosis were younger than those with primary psychiatric disorders. Approximately 30% of both groups met criteria for very early onset psychosis (<13 years) (18), but this was more frequently associated with organic causes. This relatively high rate of non-psychiatric etiologies in younger children may indicate that primary psychosis is uncommon in pre-pubertal patients (19), suggesting that careful clinical assessment may be particularly important in this age group within a tertiary setting (20).

The most prevalent diagnosis of substance-/medication-induced or medically- associated psychosis was medication-induced psychosis (7%), followed by autoimmune encephalitis (4%) and Hashimoto thyroiditis (3%). The association between autoimmune encephalitis and psychosis is well known (21–23). Neuropsychiatric symptoms related to Hashimoto thyroiditis are also common, although reports on psychotic related symptoms are less frequent (24). Medication-induced psychosis was identified in 5 cases (7%). The implicated agents included pseudoephedrine, erythromycin, stimulants, sertraline, and corticosteroids. Substance-induced psychosis occurred in one patient (1%) and was supported by a positive toxicology screen for cannabis. Notably, pseudoephedrine and erythromycin are relatively uncommon medications in the context of psychosis, although both have previously been reported to be associated with psychotic symptoms (25–27). In the medication-induced cases, symptoms resolved following discontinuation of the suspected agent.

The comparison of the psychiatric clinical presentation of our sample indicated that catatonia at first presentation of psychosis is associated with substance-/medication-induced or medically-associated psychosis diagnosis. Catatonia is a rare syndrome of abnormal psychomotor function. Its prevalence in inpatient youth varies from 0.6% to 17% (28). Published data on catatonic episodes in children and adolescents indicate that in more than 20% of cases an underlying organic condition was identified (29, 30). Some of the main etiologies of catatonia are infectious diseases, autoimmune diseases, epilepsy, and intoxication. Thakur et al. also reported similar incidence rates of catatonia cases in outpatient clinic patients; 17.7% of psychotic patients and 5.5% of all patients had catatonia (31).

Almost 60% of the patients reported hallucinations, most of them auditory. Visual hallucinations were reported by half of the patients in the substance-/medication-induced or medically-associated psychosis group, double than that in the psychosis due to a primary psychiatric disorder group; however, this was not statistically significant, probably due to the small sample size. Although visual hallucinations are common among psychotic patients, they may be a clinical sign of neurologic or ocular pathologies and therefore may be related to an underlying medical condition (32, 33).

Prior positive and/or negative subthreshold symptoms were documented in more than half of the patients and were more frequently documented among those ultimately diagnosed with a primary psychiatric disorder. These observations are consistent with prior literature suggesting that a prodromal phase often precedes the first presentation of primary psychotic disorders (34–36). Early onset, catatonia, and absence of prior documented positive and/or negative subthreshold symptoms are atypical features of psychosis as was proposed previously (11). Given that prodromal features were extracted retrospectively from clinical documentation rather than assessed using standardized instruments, this finding should be interpreted cautiously.

The medical workup showed no major differences between groups, and no laboratory tests reached statistical significance, likely due to the small number of tests per type. Autoimmune encephalitis panels were normal even in patients diagnosed with the condition, suggesting low diagnostic yield in this cohort. Ceruloplasmin was abnormal in 15% of patients, but none had Wilson disease. Since low levels can occur in non-disease states (e.g., Wilson carrier status) (37), routine ceruloplasmin testing may have limited value in similar settings, though larger studies are needed to confirm this.

There is no consensus among guidelines regarding neuroimaging in psychotic patients, although CT and MRI tests are usually requested as part of the initial medical work up (2). Previous studies report low yield of neuroimaging tests for psychotic patients, except patients with neurological symptoms or atypical clinical picture (2, 9, 38, 39). In our cohort, there were abnormal findings in the MRI test in 38% of patients: 44% in the substance-/medication-induced or medically-associated psychosis group as compared to 19% in the psychosis due to a primary psychiatric disorder group, with a significant borderline difference between the two groups. In three patients this was associated with an organic diagnosis, while the other cases had random findings with no clinical significance. This concurs with previous studies reporting that the most common findings in neuroimaging of psychotic patients are developmental abnormalities without any clinical significance (3). Although many abnormal findings were identified through medical workup, not all of them contributed to a final diagnosis. Many were deemed incidental findings, lacking clinical significance. The initial classification discussed among the authors aimed to group findings by clinical relevance, but a revised analysis approach was ultimately adopted.

The LP test is also considered a low yield test (13) in psychosis. According to our results, an abnormal LP test was not associated with higher risk for substance-/medication-induced or medically-associated psychosis. In this cohort, lumbar puncture demonstrated limited diagnostic yield and was typically performed when specific clinical suspicion was present (23).

There are mixed findings regarding the usefulness of EEG test as a screen for psychotic patient population (9, 40). This test is neither sensitive nor specific for diagnostic purposes and is often negative in people with a known epilepsy; 10% of the patients who performed an EEG test had abnormal results. Only one patient in our study was diagnosed with an epilepsy, probably related to their presented psychotic symptoms. In this cohort, EEG demonstrated limited diagnostic yield.

This study has several limitations that should be considered when interpreting the findings. First, the retrospective design introduces selection (indication) bias, and the absence of a standardized protocol for medical evaluation further limits inference. Prodromal/subthreshold symptoms were extracted from retrospective clinical documentation and were not assessed using standardized instruments; therefore, under-documentation is likely and no causal inferences should be drawn from these findings. Investigations were performed according to clinical judgment, and clinicians may have selectively ordered tests for patients in whom a secondary etiology was already suspected. As a result, the diagnostic yield reported here and the associations identified should be interpreted as hypothesis-generating rather than definitive, and their generalizability beyond our setting is limited. Second, as a retrospective chart review, the study is subject to limitations in data accuracy and completeness, and causal relationships cannot be established. The relatively small cohort limited our ability to detect rare medical causes of psychotic symptoms, and some cases of medically associated psychosis may have been missed. The small number of secondary psychosis cases also reduced the statistical power to detect meaningful between-group differences, and some associations may not have reached statistical significance as a result. Furthermore, the wide confidence intervals observed for several predictors (e.g., catatonia) indicate that these estimates should be interpreted with caution. Medical evaluations such as MRI, lumbar puncture, and EEG were not performed uniformly but rather based on clinical judgment, and the number of patients tested for certain laboratory measures was small; this selective approach could introduce bias and limits the reliability of some between-group comparisons. Because diagnostic workup and final classification were intertwined in routine care, some degree of circularity cannot be excluded. Diagnostic classification into primary versus secondary psychosis was based on clinical criteria rather than validated structured diagnostic interviews. In ambiguous cases, classification relied on multidisciplinary consensus, which, while reflecting real-world practice, may have introduced subjectivity or misclassification. In addition, abnormal test results were sometimes interpreted as incidental findings, and such judgments may vary across clinicians or institutions, potentially affecting reproducibility. The absence of neurogenetic testing may have led to missed diagnoses of genetic conditions associated with psychosis. Finally, as the study was conducted in a tertiary inpatient setting, findings may not generalize to outpatient or less acute clinical contexts. The relatively high proportion of medically associated cases (23.5%) likely reflects referral bias to a tertiary center, where more complex or atypical cases are overrepresented, and therefore the observed rate may not be representative of community-based populations.

Future research should aim to address these limitations by employing prospective study designs with standardized diagnostic protocols, which could more accurately determine diagnostic yield and inform evidence-based guidelines for the evaluation of first presentations of psychotic symptoms in children and adolescents.

### 4.1 Clinical take-home (tertiary pediatric FEP)

In our real-world tertiary cohort of children and adolescents, advanced investigations were used selectively and indication-guided rather than universally (41, 42); a small set of clinical features, particularly younger age at onset, catatonia, and absence of prior documented subthreshold symptoms, tended to accompany broader work-ups in our cohort. These observations are descriptive and hypothesis-generating and are intended to inform clinical judgment.

### 4.2 Conclusions

This retrospective pediatric FEP cohort is descriptive and hypothesis-generating. While most cases had primary psychiatric diagnoses, a subset in our setting had medical or substance-related etiologies. In routine care at our tertiary center, advanced investigations were indication-guided rather than universal. A small set of clinical flags - younger age at onset, catatonia, and absence of prior documented subthreshold symptoms, were associated with broader workups in our cohort.

## Data Availability

The raw data supporting the conclusions of this article will be made available by the authors, without undue reservation.
